# Non-Destructive Assessment of Chicken Egg Fertility

**DOI:** 10.3390/s20195546

**Published:** 2020-09-28

**Authors:** Adeyemi O. Adegbenjo, Li Liu, Michael O. Ngadi

**Affiliations:** 1 Department of Bioresource Engineering, McGill University, 21, 111 Lakeshore Road, Ste-Anne-de-Bellevue, QC H9X 3V9, Canada; adeyemi.adegbenjo@mail.mcgill.ca (A.O.A.); li.liu5@mcgill.ca (L.L.); 2 Department of Agricultural and Environmental Engineering, Obafemi Awolowo University, Ile-Ife 220005, Osun State, Nigeria

**Keywords:** chicken egg, non-destructive technologies, fertility identification, image analysis, machine vision, hyperspectral imaging

## Abstract

Total hatching egg set (for both egg production chicks and broilers) in the Agriculture and Agri-Food Canada report 2017 was over 1.0 billion. With the fertility rate for this year observed to be around 82%, there were about 180 million unhatched eggs (worth over 300 million Canadian dollars) incubated in Canada for the year 2017 alone. These non-hatching (non-fertile) eggs can find useful applications as commercial table eggs or low-grade food stock if they can be detected early and isolated accordingly preferably prior to incubation. The conventional method of chicken egg fertility assessment termed candling, is subjective, cumbersome, slow, and eventually inefficient, leading to huge economic losses. Hence, there is a need for a non-destructive, fast and online prediction technology to assist with early chicken egg fertility identification problem. This paper reviewed existing non-destructive approaches including ultrasound and dielectric measurements, thermal imaging, machine vision, spectroscopy, and hyperspectral imaging. Hyperspectral imaging was extensively discussed, being an emerging new technology with great potential. Suggestions were finally proffered towards building futuristic robust model(s) for early detection of chicken egg fertility.

## 1. Introduction

Over 50 billion chickens are being raised annually by poultry farmers all over the world, be it as layers towards egg production or as broilers towards meat production, and production growth is anticipated to continue. The global world population has been projected to hit 9.6 billion by 2050, creating an increasing demand for animal-based food [1]. Even though pork and beef demand could increase by up to 43% and 66% respectively, poultry meat has been projected to have the greatest growth rate of up to 121%, and demand for eggs is expected to increase by 65% [2]. For the year 2017, Canada exported over 39 million hatching eggs (worth over $68 million), with the US being the largest market. The importation figure for the same year however stood at over 141 million hatching eggs for broilers (worth over $49 million), with entire importation coming from the US (Agriculture and Agri-Food Canada report 2017).

Seeing the great importance of chicken and chicken eggs both locally and globally, it is imperative especially in the present era of advancing technologies in the field of machine learning and artificial intelligence, that there would be worthwhile assistance towards improving the hatchability rate of chicken eggs. Early fertility (prior to incubation) and/or embryonic development detection would prevent wastage of egg and incubation energy, make more incubator space available for viable hatching eggs, and likewise promises huge economic returns. Achieving the above would be a crucial component of the effort on achieving the sustainable development goal 2 (SDG2) agendum.

### 1.1. Egg Formation and Structure

**Figure 1:**
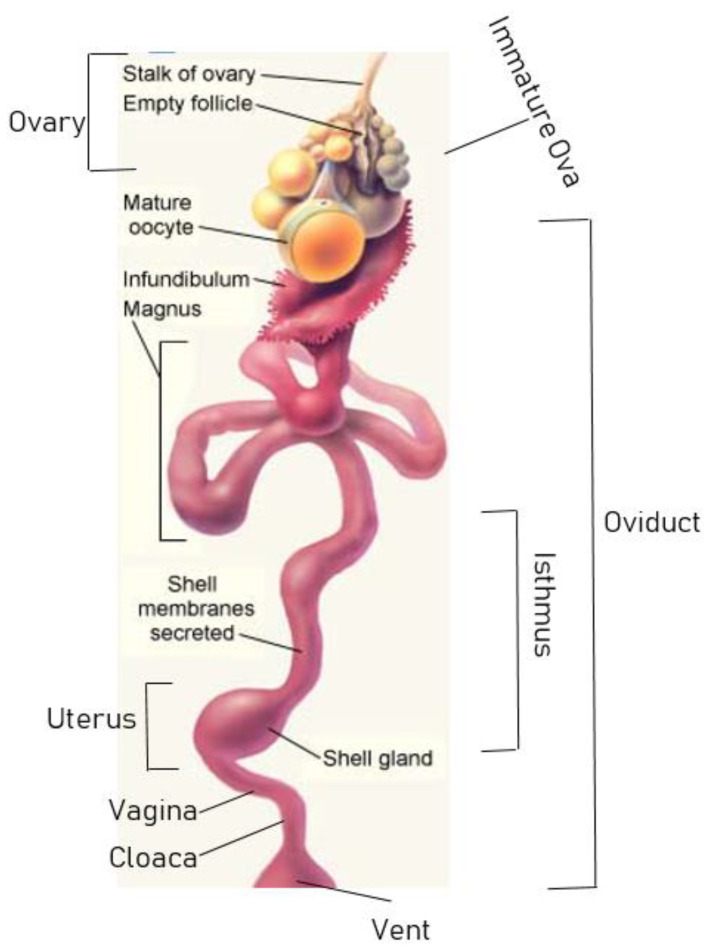
Schematic diagram detailing the process of egg formation.

Egg formation is a process that occurs in about 25–26 h from ovulation to oviposition. It commences with a matured ovum (which is a plain yolk and germinal disc) in the reproductive tract, resulting at last in a hard shelled egg, fully complete with its own protective membranes and the necessary nutrients needed for embryonic development [3]. The major stages in egg formation include the ovulation stage, fertilization stage, formation, and oviposition. All these stages are accomplished in the ovary and the oviduct. The process can be better and clearly understood considering the schematic diagram in Figure 1.

In the ovary, the ovum or oocyte is released from the follicle through a process known as ovulation. Ovulation takes place in about 5 to 10 min following the expulsion of the previous egg. This stage has been preceded with yolk production made possible from the chickens (hens) being fed with diets containing appropriate nutrients. Diets rich in calcium are of good necessity at this stage as it will find useful application later during the shell formation. These nutrients absorbed into the bloodstream from the hens’ digestive tracts are converted into yolk by the hen’s liver. The yolk is then transported through the blood stream from the liver to the ovary. Here, the follicular cells around the ovum take the yolk and other nutrients and carry them along to the ovum. The immature ova and their neighboring follicular cells are securely embedded within the ovary. As the ovum increases in more and more yolk accumulation, it becomes greatly enlarged so that it can no longer fit inside the ovary. Therefore, there begins a gradual continuous pushing of the nested ovum and the ovarian follicle towards the outer ovarian edge.

As the ovum accumulates enough yolk that is adequate for growing a chick, the ovum ruptures from its follicle through a process earlier mentioned as ovulation. The free ovum drops into the ovarian pocket and within minutes is captured by the infundibulum and is guided into the mouth of the hen’s left oviduct. Almost immediately the ovum is released from the ovary and before being received by the infundibulum, the egg’s nucleus passes through a process of primary cell divisions known as meiosis and it is only one of the cells (others fade away naturally) produced from meiosis, which ends up becoming a matured ovum that is accepted by the infundibulum. Fertilization occurs inside the infundibulum if sperm is available, and the resulting zygote thereby commences a secondary cell division via mitosis. The first layer of albumen is also deposited at this stage [4]. The remaining process of egg formation is completed during the journey down the oviduct.

**Figure 2:**
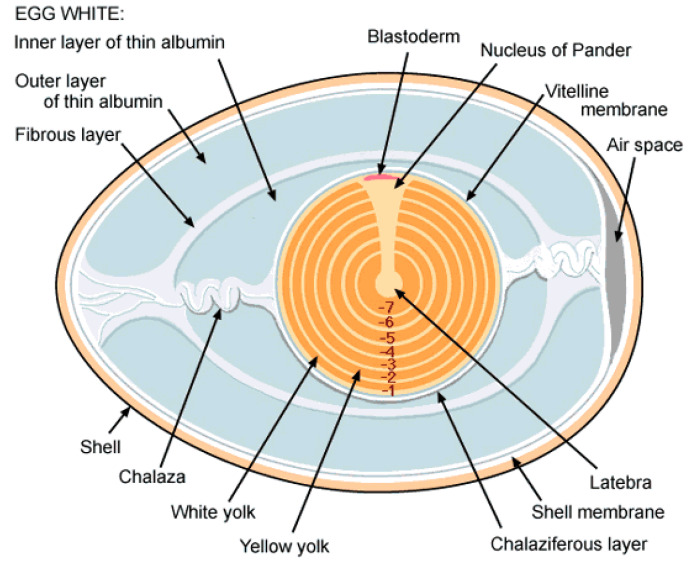
Chicken egg structure.

The oviduct is divided into six different sections which are: infundibulum (oviduct’s mouth or funnel), magnum, isthmus, shell gland (uterus), vagina, and the cloaca. Whether the ovum is fertilized or not (as in the case of table or hatchery infertile eggs), it continues its journey along the oviduct to allow for complete covering by layers of egg white (albumen) and other internal supporting structures. The section of the oviduct responsible for most of albumen secretion is the magnum. The matured ovum and its surrounding layers reaching the magnum can now at this point be conveniently called an egg (if fertilized, an embryo is formed). Due to the spiral structural design of the oviduct, the egg twists/rotates along its journey and some protein fibers extension from the egg are hooked by the thick and thin albumens secreted along the oviduct. This occurrence results in albumen layers and chalazae formation. The shell membranes are then added in the Isthmus and the shell gland located in the uterus later commences the process of shell formation [5]. The average times, as reported by [6,7], an ovum spends in each section as it travels down the oviduct are: infundibulum 15 min, magnum 2–3 h, isthmus 1 h, uterus 21 h, and vagina/cloaca just a few minutes. A finally formed whole egg structure as shown in Figure 2 consists of 30–33% yolk, about 60% albumen, and between 9–12% shell [8,9].

The eggshell is deposited while the egg is still in the hen’s uterus. Three distinct stages of eggshell formation can be identified according to Hernández-Hernández, et al. [10] namely: (a) initial, (b) fast growth, and (c) termination. The initial stage begins with calcium carbonate (CaCO_3_) spheruliths forming on the eggshell membranes. This formation progresses until adjacent spheruliths are knitted (fused) together, a process known as nucleation. After this is an emergence of columnar crystals (palisades) from the spherules during the fast growth stage. Columnar crystal formation continues until eggshell calcification is completed with the deposition of the cuticle layer in the termination stage. It is important to mention that for brown eggs, deposit of protoporphyrin (pigment responsible for the brown colouration) occurs both at the onset and termination stage of shell formation.

### 1.2. Chicken Egg Chemical and Functional Compositions

The average weight of chicken egg is around 58 g, comprising various components including up to 11% lipids, 12% protein, and 74% water [11]. The yolk forms between 30–36% of the total fresh whole chicken egg weight, with dry matter content of a freshly laid yolk being between 50 to 52% depending on the age of the laying chicken [12].

According to a scientific report by the University of Illinois at Urbana-Champaign, USA [5,13], the yolk during its last 7 to 8 days of development is deposited in ring-like layers of white and yellow yolk. These ring-like layers though mostly not visible at earlier days of yolk formation is usually visible under MRI image in the last days of yolk formation. Important features can be typically found in an MRI image for earlier and last days of yolk formation. Whereas the yellow yolk (rich in lipids or fats, and deposited during the day) appears dark in MRI image, the white yolk (abundance in protein, and deposited during the night) is commonly seen as narrow white bands on the MRI image [4]. The white yolk was further reported to be present directly below the nucleus (position of potential future embryo development) in the latebra and the nucleus of pander and observed to be arranged concentrically all over the yellow yolk (see Figure 2). The existence of yellow and white yolk has earlier been attested to in literatures [14,15], where it was reported that the white yolk originated from the maturing white follicle in the ovary. It was also pointed out that several structures like the latebra, nucleus of pander, and embryonic disc all originated from the white yolk and that the embryonic disc in the nucleus of pander is the position for embryonic development.

**Figure 3:**
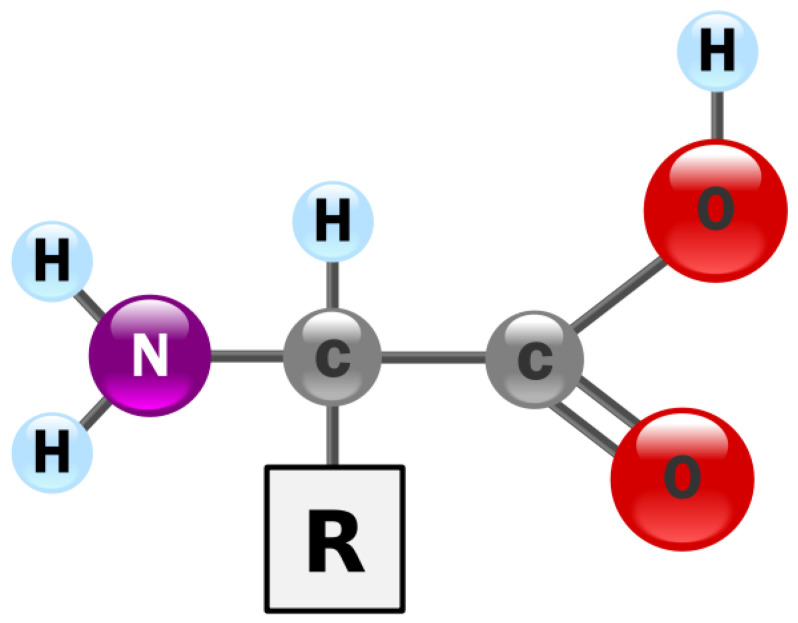
Amino acid structure showing its various functional groups.

The understanding of the yellow and white yolk, together with its constituents and positioning in the whole egg, carries a great potential of assisting towards developing a more targeted approach for early chicken egg fertility assessment. There are 22 genetically encoded (proteinogenic or protein creating) amino acids, of which 18 present in chicken egg yolk, albumen, and whole egg, are as listed thus: Alanine, Arginine, Asparagine, Cysteine, Glutamine, Glycine, Histidine, Isoleucine, Leucine, Lysine, Methionine, Phenylalanine, Proline, Serine, Threonine, Tryptophan, Tyrosine, and Valine [11]. Amino acids, as shown in Figure 3, can be described structurally as organic compounds with two functional groups namely amine (-NH_2_) and carboxyl (-COOH), together with a side chain -R group (specifically related to each amino acid).

Considering the white yolk regions for in-depth analysis and targeting the protein constituents in chicken egg yolk can open a new door for research opportunities towards identifying specific biomarkers for differentiating between fertile and non-fertile eggs prior to incubation.

## 2. Chicken Egg Fertility and Incubation

Chicken eggs are said to be fertile if the hen that laid the eggs was raised together with roosters, otherwise the eggs would be infertile. Fertilization is established from the unison of the rooster sperm with the hen’s matured ovum, be it naturally or artificially through a process known as artificial insemination. While the majority of the global hatchery (fertile) eggs are mostly produced from a mother hen raised together with roosters, grocery store (table) eggs are from hens raised without roosters. It is only fertile eggs that carry embryonic development potentials under incubation conditions of around 55% relative humidity and temperature of 37.8 °C. Notwithstanding, it is not all the so-called fertile eggs that end up becoming chickens under incubation conditions, as some indeed eventually do turn out to be non-fertile eggs. Hence, there is a need to know and understand the difference between hatchery fertile and non-fertile eggs.

**Figure 4:**
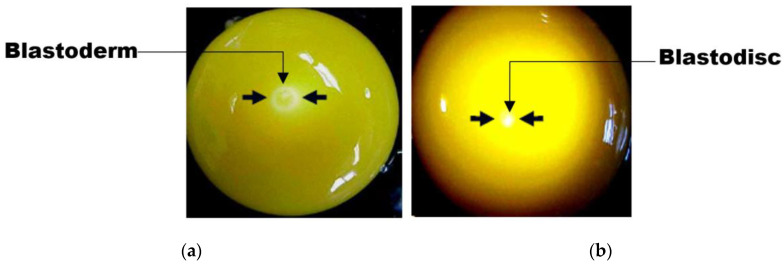
Chicken egg fertility identification: (**a**) blastoderm and (**b**) blastodisc.

According to [16,17], fertilized eggs contain blastoderm, while unfertilized eggs contain germinal disc (blatodisc). The blastoderm (in a fresh opened egg) is visually seen as a symmetrical circular ring of about 3–4 mm in diameter (Figure 4a), having a less-dense “Area Pellucida” and denser “Area Opaca” regions around its perimeter. The blastodisc in comparison to the blastoderm has a smaller diameter (about 2.5mm) and looks like an asymmetrical solid spot (Figure 4b), with no regional differentiation. Under a stereomicroscope, the Area Pellucida (AP) and Area Opaca (AO) are clearly differentiated in the fertile egg blastoderm. On the other hand, the germinal disc visualization on a stereomicroscope is usually characterized by many vacuoles (bubbles), anciently known as lacunae [15].

## 3. Industrial Challenge

**Figure 5:**
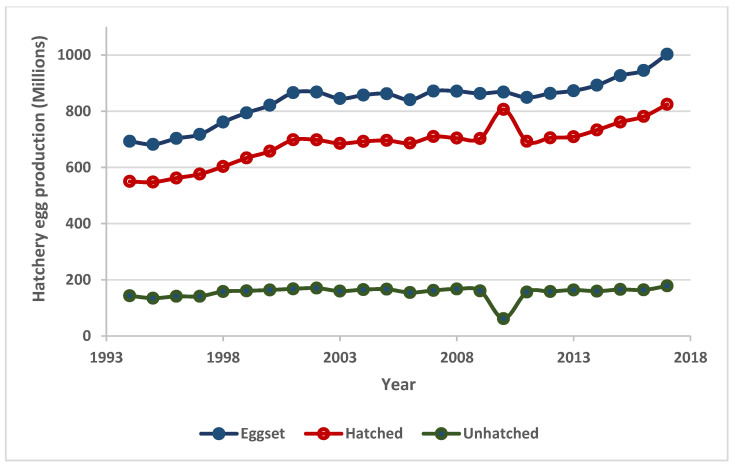
Canadian hatchery egg production from 1994 to 2017.

**Figure 6:**
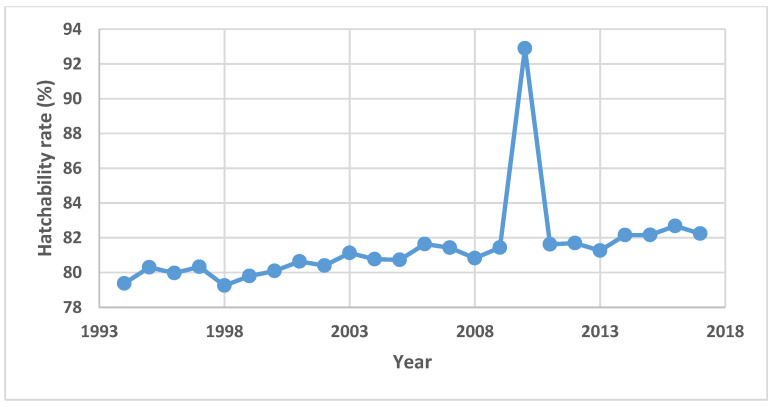
Hatchability rates for Canadian egg production.

Complete hatchability of incubated eggs remains of a great economic concern to poultry farm owners globally. Several factors including but not limited to environmental and genetic factors have been known to cause decline in chicken egg fertility and hatchability [18]. While some of these factors could be arrested prior to egg production and incubation, some are only traceable after the havoc is already done. Hence, fertility and hatchability rates continue to dwindle from year to year resulting in losses of millions of dollars annually. Figure 5 showed the total hatchery egg (both for layers and broilers) production in Canada for the years 1994 through 2017. Apart from the year 2010, where there was a sudden jump in the number of hatched eggs, with a corresponding decline in the number of unhatched eggs, other increases in the amount of hatched eggs over the years have been relative to corresponding increases in the total number of eggs set available for incubation. It was therefore observed that the amount of unhatched eggs over the years have not reduced, having its least of over 134 million in the year 1995 and its peak of over 178 million in the recent past year 2017. With about 39.8 million eggs sold in the year 2017 via exportation to the United States at a sum of about 68.8 million dollars, over 300 million dollars’ worth of eggs were wasted as unhatched eggs in 2017. Except for year 2010, where hatchability rate stands at about 93%. Figure 6 showed that hatchability rates over all other years between 1994 and 2017 have not seriously improved, being in the range of 79% to 83%. Therefore, it is very critical to determine fertility and viability of chicken eggs prior to incubation. A fast, online, and non-destructive pre-screening of eggs for fertility identification before being passed for incubation would save industries both immediate and impending losses.

## 4. Assessment of Chicken Egg Fertility

### 4.1. Conventional Methods

**Figure 7:**
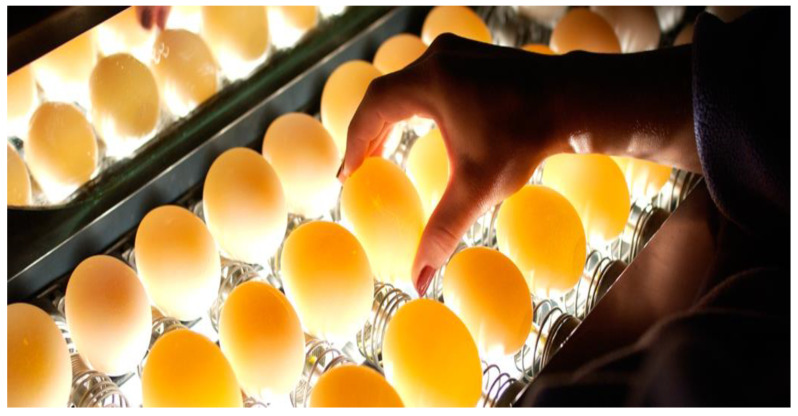
Traditional candling operation.

Conventional methods for determining chicken egg fertility are cumbersome and destructive in nature. These approaches include the breakout fertility (fresh breakout or candled breakout), visual and microscopic approaches, sperm counting in the outer perivitelline layer, and the sperm penetration assay technique [19]. The candling procedure in the breakout fertility approach is the most popular and is as depicted in Figure 7. Not only is this method slow and labour intensive, but also about 5% of the whole egg set are randomly candled on day 10 while the remaining 95% are left to chances. In the long run, a larger percentage of non-fertile eggs ends up being incubated, which usually exposes the whole egg set to contamination in the case of exploder eggs. Not only that, millions of dollars ended up being lost every year as a result of various bottlenecks [20,21], including incubation space, energy, and egg wastages. Furthermore, these methods are not appropriate for building an online egg fertility classification system in the fast-paced technology advancing era of the day. Hence, there is a need for a robust, non-destructive, fast, and online prediction technology to assist with early chicken egg fertility identification problems.

### 4.2. Non-Destructive Methods

Various non-destructive methods including measurement of ultrasound signals and dielectric characteristics, thermal imaging, machine vision, spectroscopy, and hyperspectral imaging have been harnessed for assessing chicken egg fertility. Prominent approaches, however, are the machine vision, spectroscopy, and hyperspectral imaging. Önler et al. [22] studied fertility discrimination of brown eggs using ultrasonographic images. Even though model accuracy of up to 86% was presented, it was pointed out that the ultrasound signal could not penetrate the eggshell and so holes needed to be created on the eggs before acquiring the ultrasound signals. In the work of [23], dielectric properties and intelligent methods were considered for classifying hatching eggs during incubation. Model accuracy of 100% was recorded on day 18 incubation for dead embryo with SVM classifier. Lin et al. [24] experimented on using thermal imaging system for filtering and recognition of fertilized eggs. The accuracy reported was 96% for 14 days incubated eggs. These incubation days (18 and 14 respectively) are, however, too late for early identification desired in the poultry industries. Due to the recent advancement in data acquisition, management, storage and archiving, the practice of incorporating machine and deep-learning architectures in non-destructive technologies application has been on the increase; apart from the conventional multilayer perceptron (MLP) feed forward artificial neural network (ANN), other deep ANN (DNN) that have found increasing applications in the analysis of hyperspectral imaging data are deep belief networks (DBNs), autoencoders (AEs), recurrent neural networks (RNNs), and convolution neural networks (CNN) [25,26]. Geng et al. [27] described a non-destructive method involving transfer learning procedure and convolution neural network (CNN), with reported good model generalization capability and egg diversity adaptation. This study, however, was not explicit about the source and nature of the images used. Likewise, it was not stated clearly how the different classes of fertile, infertile, and dead embryo eggs was determined. In the same vein, a follow up study to [27] by [28] adopted a dense pixelwise spatial attention (DPSA) network for hatching egg fertility classification. The same methodology as in [27] was followed and so the questions regarding image data source and classes determination remain unanswered. Understanding the great influence of data quality on predictive modelling outputs, this kind of gap is very critical to fill for reproducible research and towards building robust models. Non-destructive spectroscopy and hyperspectral imaging methods have some specific data analysis steps including spectra processing, image processing (including but not limited to image enhancement and textural feature extraction), model establishment, and chemical imaging visualization [29,30]. The modelling procedure also entails the use of various algorithms namely image/spectra calibration, preprocessing, wavelength selection, regression, and classification algorithms [30]. A discussion of more popular non-destructive approaches is presented in the following sub sections.

#### 4.2.1. Machine/Computer Vision

Great advancement has been achieved over the years for safety inspection and quality sorting of agricultural and food produce [31]. According to [32], machine vision technology adopts image processing and analysis procedures, in combination with a set up including illumination system and personal computer connected to a form of mechanical or electrical device. Das and Evans [33,34] used machine vision to recognize fertility of hatching eggs in conjunction with histogram characterization and neural network methods. Obtained accuracies for the work were low at early days of incubation but high on days 3 and 4 incubation. These results might be attributed to the limitations of machine vision approach ranging from inability to detect intrinsic properties and to handle difficult classification tasks [31,35].

Since various food analysis situations exist, necessitating acquiring information from inside the sample, rather than from the outside, machine vision might not be the best appropriate technology for early chicken egg fertility detection. Also, because acquisition and analysis are usually done using the three RGB spectra channels in the visible wavelength range of the electromagnetic spectrum [36], it deprives users the advantage of benefiting from considering a wider range of wavelength bands like those in the near, mid and far infrared regions.

#### 4.2.2. Spectroscopy and Hyperspectral Imaging

Both mid-IR and NIR spectroscopy are similar based on their fundamental principles of operation, which entails a consideration of molecular vibrations [37,38]. However, due to different excitation conditions of both mid-IR and NIR spectroscopy and depending on the product’s compounds of interest, the relationship between the functionalities of the molecules being examined and the corresponding absorption intensities differ considerably, thus leading to a significantly different responses of the same molecular vibration, from the same applied fundamental technique [38]. Mid-IR spectroscopy involves mostly fundamental vibrations and is found in wavelength regions between 2500 and 25,000 nm (4000–400 cm^−1^) of the electromagnetic spectrum. NIR spectroscopy on the other hand entails radiations that are higher than that in mid-IR and found in the wavelength regions between 800 and 2500 nm (12,500–4000 cm^−1^).

NIR spectroscopy is among the most popular in the food industry. The absorption bands viewed in the NIR region are from overtones and combination bands of C-H, N-H, O-H, and S-H bending and stretching vibrations. Hence, the NIR technologies are applicable to all organic compounds abundant in C-H bonds (like petroleum derivatives), O-H bonds (like carbohydrate, moisture, and fat), and N-H bonds (like amino acids and proteins). Since all biological substances consist of numerous amounts of O-H, N-H, and C-H molecular bonds, NIR radiation striking a sample, produces a multiplex spectrum carrying both quantitative and qualitative information about the specific sample [31].

Visible (VIS) and Near Infrared (NIR) spectroscopy have been widely utilized in assessing internal quality of Agricultural products [39,40,41]. Norris [42] studied the effect of storage on optical properties of shell eggs in the NIR region. Even though progressive changes were noticed in the spectral data during storage periods, it was further observed that there was no correlation between these changes and internal egg quality indices, thus necessitating further researches and/or improvement in existing technology to make this non-destructive approach more relevant for industrial applications. Bamelis et al. [43] adopted a spectrophotometric method known for blood detection in Table eggs to assess early embryonic detection potential in fertile chicken eggs. The work reported embryonic development detection to be possible from day 5 (120 h) of incubation. This late detection might be partly related to the point information acquisition mode of spectroscopy technique, making it disadvantageous when information of interest is not present in the pixel spot measured. This disadvantage is catered for in the hyperspectral imaging technology. A recent study by [44] used visible near infrared transmission spectroscopy in conjunction with Naïve–Bayes classifier for infertile chicken eggs prediction before hatching. The average overall prediction accuracy, specificity, and sensitivity values were 89.12, 90.38 and 88.14% respectively. As much as these results were promising, it was observed that the fertility status of all the considered egg sets was not conventionally confirmed following standard incubation conditions. Notwithstanding, the study noted that accurate prediction was possible only in the spectral range 500–940 nm excluding data in the spectra ranges 330–500 nm and 940–1030 nm, due to low signal-to-noise ratio and low absorption characteristics. It can then be inferred from this observation that spectra data in the higher NIR region above 1030 nm might carry better discriminative information.

Hyperspectral imaging being an improvement on conventional spectroscopy and machine vision has witnessed a wide publicity in recent times due to its combined ability to consider both spectra and spatial information from targeted samples [45,46]. The near-infrared (NIR) regions in particular have been successfully used for food (including chicken egg) quality and safety analysis [39,41,47,48,49] and could as well prove effective for chicken egg fertility assessment studies. Hyperspectral imaging, though a relatively new and emerging technology, has proven more advantageous than spectroscopy and computer vison due to its chemical-free assessment, non-destructive and non-invasive nature, spatial distribution visualization, fast image acquisition potentials, little or no sample preparation, eventual simple and fast analysis method, flexibility in region of interest (ROI) selection [31,50], and ability to handle sample heterogeneity.

## 5. Hyperspectral Imaging Technology and Instrumentation

**Figure 8:**
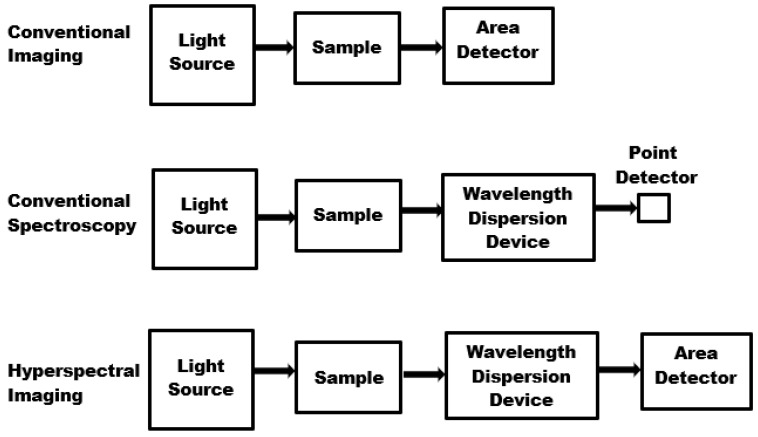
General system configurations for conventional imaging, conventional spectroscopy, and hyperspectral imaging.

Optical sensors have been known to provide great potentials for non-destructive analysis of agricultural products. Imaging and spectroscopic techniques have been widely studied and used in various fields of endeavours including agricultural applications [43,51]. However, spectroscopy and conventional imaging approaches are limited when it comes to obtaining adequate information from individual food items [52]. Due to recent advancement in imaging and spectroscopy technologies, hyperspectral imaging has emerged as a preferable alternative for quality assessment and safety control of agricultural produce [53,54,55]. Hyperspectral imaging instruments can measure signals from the various regions of the electromagnetic spectrum including but not limited to the visible (VIS), near infrared (NIR), and mid infrared (MIR). Some other hyperspectral imaging instrumentations of increasing attention are the confocal laser microscopy scanners, Raman spectroscopy, Terahertz spectroscopy, X-ray spectroscopy, magnetic resonance imaging, and 3D ultrasound imaging [56]. Generally, a hyperspectral system comprises of a light source, a wavelength dispersion device, and an area detector. Figure 8 showed a schematic description of the emergence of a hyperspectral imaging system from conventional imaging and spectroscopy [52]. The choice of illumination source is a very critical factor of consideration in the planning and setting up of any imaging system. For example, the nature of the emitted spectrum and the amount of light intensity reaching the object of interest will influence the subsequent quantity of light absorbed, transmitted, or reflected from the object. Hence, only the wattage rating of lamps is not enough in selecting a suitable light source for imaging application, but is for the illuminance of the said light source. Some lamps with higher wattage ratings have been shown to have lower illuminance when compared to other lamps of lower wattage ratings. This is because a larger percentage of light emanating out of some lamps is being lost as heat and, therefore, is unusable. According to Qin [52], halogen lamps are the most popular broadband illumination sources that have been successfully used in the visible (VIS) and near-infrared (NIR) wavelength regions for hyperspectral imaging. Specific applications include but are not limited to “pits detection in tart cherries”, “bone fragment detection in chicken breast fillets” and “detecting fertility and early embryonic development of chicken eggs” [57,58,59]. Other illumination sources with the potential of gaining wide acceptability in the nearest future are lasers, tunable sources and light-emitting diodes [60,61,62,63,64,65,66,67,68].

### 5.1. Principle of Operation

**Figure 9:**
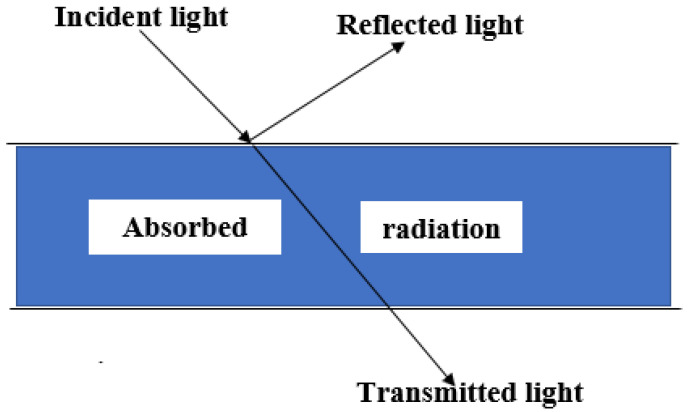
Hyperspectral imaging principle of operation.

**Figure 10:**
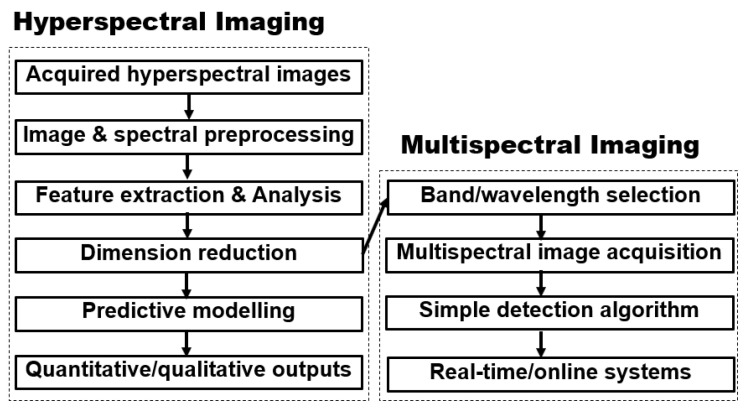
Hyperspectral-Multispectral imaging flow chart.

Hyperspectral imaging works on the optical principle of light and its interaction with matter. When light energy (photon) falls on an object, you do not see the light, but the amount of light energy available determines how much of the object you see based on the influence of the light. The brain behind hyperspectral imaging system as a tool for non-destructive food analysis is therefore based on the understanding of light photons interaction with the molecular structures of food samples [31,69]. As light energy strikes an object, the incident light reaching and interacting with the object can be reflected, absorbed, or transmitted as depicted in Figure 9. These reflected, absorbed, or transmitted lights carry important information from the passing medium (object) and can be used for both qualitative and quantitative predictions. Figure 10 showed the summarized basic steps involved in hyperspectral imaging analysis, with an overall intention of being translated into a multispectral imaging system, which is usually more economically built and suitable for an online and real-time industrial application [70].

### 5.2. Image Acquisition, Data Extraction, and Spectra Pre-Processing

Knowing well that it is practically impossible to get any useful information from less qualitative data, obtaining a high-quality image therefore becomes very critical in hyperspectral imaging and related researches. Consistency and accuracy are needed in various settings including acquisition mode, illumination type and arrangement, detector selection, spectral and spatial resolutions, frame rate, scanning speed, camera exposure/integration time, and calibration [36,71,72]. For a typical hyperspectral image, a continuous spectrum for individual pixel is usually measured, with spectral resolution presented in wave numbers (cm^−1^) or nanometers (nm). A hyperspectral image is usually displayed as a hypercube in three dimensions including two spatial (X, Y) and one spectral (λ) dimension. This hypercube structure contains chemical information related to the target of interest [56,73].

After image acquisition, spectral information (X-matrix) is usually extracted from a segmented region(s) of interest (ROI’s). This is a very crucial step before further sample analysis, especially when the targeted portion does not consist of all the scanned area. This step saves the whole modelling process a huge amount of computing time [74]. The ROI’s stand for expected or actual locations of targeted biological or quality attributes in the acquired image [75,76]. A corresponding response or reference information (Y-matrix) is also collected following the standard conventional (usually destructive) method. The response information is ideally obtained at the exact ROI’s from which the spectra (X-matrix) data has been collected [36]. Depending on the degree of quality achieved in the acquisition/extraction stage, hyperspectral images/data usually contain noise and some unwanted variabilities due to various other factors including but not limited to detector anomalies, particle size variations and light scattering effects. Hence, spectra pre-processing is usually implemented to minimize the effect of the above-mentioned problems. Spectral pre-processing can consist of one or a combination of the following techniques namely: filtering, smoothening, normalization, mean centering, scaling, standard normal variate (SNV), multiplicative scatter correction (MSC), orthogonal signal correction (OSC), derivatives, detrend, Fourier and Wavelet transforms [77,78,79,80].

Filtering is purposely employed to remove non-informative variables in a data set. This approach eventually results in improved statistical power during a downstream multivariate analysis. Smoothing filters on the other hand are usually implemented to reduce noise, while simultaneously preserving the number of variables [81,82]. Normalization is used in adjusting samples to approximately the same scale. The most common approach is the use of mean centering (dividing each instance of a data matrix by its mean) or autoscaling (mean centering + division by standard deviation of individual variable). Other forms of normalization like normalization by sum, median, reference sample, reference feature and data transformation such as logarithmic and cube root transformations are used for general purpose modification for variability among instances and to make individual attributes more comparable [81,83]. According to [83], MSC and SNV are used to adjust for multiplicative and/or additive effects in spectra data, including removing particle size effect and correcting for path length variation. Detrending, used for removing nonlinear drifts in spectroscopic data, is also versatile in reducing data baseline shift, curvature, and multicollinearity, when implemented in conjunction with SNV. Derivatives of various orders are row-oriented transformations widely used to reveal hidden information that might not be visible considering the raw data spectrum. Recently, [84] proposed a data-driven learning approach to tackle the challenge of spectral variability in hyperspectral unmixing, The technique was reported to model main spectral variability (e.g., scaling issues from illumination/light scattering effects) as separate from other variabilities (including environmental conditions, detector anomalies, etc.), using endmember and spectral variability dictionaries. Most of the pre-processing procedures as iterated above are commonly implemented with the aid of specialized software packages including Unscrambler, Matlab, WEKA, SAS, JMP, and other related packages.

### 5.3. Dimensional Reduction Techniques

Since HSI sensors produce a great number of spectral bands, the major task in HSI analysis entails dealing with a huge amount of data. This does not only increase computational difficulties but also in the long run severely affects classification accuracies. Hence, various feature extraction techniques are usually employed to reduce dimensionality and extract important wavelength bands from HSI data, thereby eliminating as much as possible spectra redundancy from the acquired multidimensional HSI data [85]. Band selection approaches have been grouped into six namely ranking-based, searching-based, clustering-based, sparsity-based, embedding learning-based, and hybrid scheme-based methods [86]. Graph learning dimension reduction method has also been reported as an effective approach for analyzing intrinsic characteristics of hyperspectral imaging data [87]. The most widely used dimensionality reduction techniques, however, are the linear methods of principal component analysis (PCA) and multidimensional scaling (MDS). Others include but are not limited to partial least square (PLS), ISOMAP and Autoencoder [88,89,90]. Some of the existing dimensional reduction techniques can select important bands and simultaneously extract new wavelength bands for discrimination, and this is the reason some researchers do mistakenly accept feature band extraction and band selection to be the same. There exist indeed other specific approaches solely for bands selection. Whereas bands extraction creates new attributes (transformed wavelength bands) from functions of the original wavelength bands, band selection chooses a small subset of the original wavelength bands set, which performs optimally under some criterion function. Band selection has been widely accepted as appropriate for hyperspectral imaging data due to its aid in speedy information acquisition/processing and eventually resulting in great cost savings [91].

During band selection, the choice of subsets per time to be learned are usually determined using the embedded, filter and/or wrapper methods [92,93]. While the filter approach considers the appropriateness of the selected bands, independent of the classifying algorithm, the wrapper method requires a classifier to evaluate wavelength band appropriateness, but also can be computationally burdensome. Although the filter techniques are classifier-independent, simple and fast, they are limited due to their dark knowledge of the interaction between band subset search and classifier [94]. This disadvantage with the filter techniques is catered for in the wrapper and embedded methods. Also, there exist multivariate filters purposely developed to overcome the limitation of the conventional filter approach, and these include the information gain, correlation and learner-based band selection techniques [94,95,96]. Whether it be filter, wrapper, or embedded-based band selection system, their implementation is always in conjunction with various search algorithms. Such algorithms according to the work of [91] have been described in terms of optimal, quasi-optimal, and ratio band selection algorithms.

A wavelength band selection algorithm is said to be optimal if it chooses the best subset of “m” of “n” attributes, with the best “m” determined as being the subset having maximum value for a chosen criterion function. While the optimal algorithms include the exhaustive search and the branch-and-bound (BB) algorithms, quasi optimal algorithms include the sequential forward floating (SFFS) and sequential backward floating (SBFS) selections [97,98,99]. The choice of selection algorithm is greatly dependent on the number of “m” subsets to be evaluated per time for criterion function computation. The greater the number of criterion functions to be computed, the greater the search time will be, and this is invariably dependent on the total number of original “n” bands. For example, an exhaustive search for the four best wavelength band attributes from a 400-featured data set will need a search of “400 combination 4” (^400^C_4_) ≈ one billion subsets [91]. This search is known to be exponentially increased, should there be a consideration of ratio wavelength band selections.

### 5.4. Ratio Band Selection Algorithms

Ratio wavelength bands have been reported as having better discrimination potentials than an individual wavelength band. Guyon and Elisseeff [100] indeed confirmed experimentally that a wavelength band attribute that is completely useless alone, can provide notable performance improvement when considered alongside other wavelength bands. It was also buttressed in the same work that two wavelength bands that are redundant by themselves can be useful together. Hence, ratio wavelength bands consideration has begun to gain increasing interests in band selection approaches. Xia and Wishart [81] reported the use of a PLSDA learner and ranking algorithm to select up to 100 ratio attributes in an online metabolomic analysis platform. Ideally, depending on the number of original “n” attributes, there could be numerous ratio attributes to be computed. For example, there would be “167 combination 2” (^167^C_2_) = 13, 861 possible ratio attributes out of 167 original wavelength attributes. To choose only two best sets of ratio attributes from the above will require exhaustive search to calculate criterion functions for all (^13,861^C_2_) ≈ 96 million combinations of two sets of ratio attributes. Since these numbers of combinations can greatly increase exponentially depending on original “n” attributes and “m” subsets of ratio attributes needed, quasi-optimal algorithms including SFFS and SBFS have been suggested feasible for large ratio attributes computation and eventual ratio subset selections. Nakariyakul [91] suggested a new adaptive branch and bound (ABB) algorithm, an improved sequential forward floating selection (ISFFS) algorithm, and a fast ratio attribute selection algorithm, which were all successfully tested on chicken skin tumor and chicken contaminant hyperspectral imaging data.

Even though there have not been many works on using ratio attributes in chicken egg fertility studies. Bamelis et al. [43] reportedly used the ratio wavelength 527/610 nm, popularly used in commercial blood detectors for early embryonic development detection in chicken eggs. The work, however, concluded that embryonic development detection with visible light transmission is not directly correlated with blood formation. Therefore, there is a need for more studies to consider many possible ratio attributes from chicken egg fertility data, before arriving at optimal ratio attribute selections.

### 5.5. Multivariate Analysis (Post Processing)

Multivariate analysis (MVA) can be categorized into three major areas namely: exploratory data analysis (EDA), regression analysis and discrimination analysis (classification). EDA is also often called data mining and it is the common approach used towards understanding deeper insights into large and complicated data sets. While regression analysis assists in model development towards prediction of new and future events, classification on the other hand is a versatile tool useful in research, development, and market analysis, towards handling categorical data. Even though each method of MVA used on its own can produce worthwhile results, effective combination of these methods can bring about outstanding revelations about the system under study [101]. Two main approaches commonly employed in EDA are cluster analysis and principal component analysis. Whereas cluster analysis achieves the job of isolating objects into groups (clusters) in which members of an identified cluster are related to each other, PCA analyses variability in data set, towards reducing dimensionality of large data. Understanding correlations between samples and variables is usually of critical consideration in PCA.

#### 5.5.1. Regression Analysis and Predictive Modelling

Regression models are models used to predict numeric outcome and are therefore also called quantitative models [102]. Regression analysis produces only continuous responses. According to Swarbrick [101], regression analysis often involves two data sets comprising of the predictors (independent) and dependent (response) variables. Independent variables are already known measurements to make models from, towards predicting the required output. Dependent variables on the other hand are the responses being modelled from the predictors. Responses depend greatly on the predictors used in the model. Widely used multivariate regression methods include multiple linear regression (MLR), principal component regression (PCR), and partial least squares regression [36,103]. In the situation of non-linearity expectation (resulting from signal artifacts) in which the Beer–Lambert law is not followed, methods such as support vector regression (SVR) and artificial neural network regression (ANN-R) have been reported as good alternatives for modelling HSI data. It is, however, appropriate to consider various preprocessing methods in addressing deviations from linearity before adopting SVR and/or ANN-R [77,104,105].

#### 5.5.2. Discrimination Analysis/Classification Algorithms

Discrimination is the term used to describe separation (or division) of a group of samples into one or more classes based on characteristic features in the samples. Discrimination (classification) has been known to be a very crucial task in pattern recognition. Discriminative models are also known as qualitative or categorical response models. Two basic approaches to solving classification problems in general are those of unsupervised and supervised algorithm techniques. In unsupervised learning, data are grouped based on some similarities/dissimilarities or characteristics inherent in the data set, and the analyst may not have a priori knowledge about the grouping. Supervised learning on the other hand gives the opportunity of having the idea of what factors, input or predictors will have an impact on the output response even though one might not have the complete understanding of the relationship between the response and the predictors. Notable methods used in unsupervised classification include K-means, K-medians, hierarchical cluster analysis, and principal component analysis. For supervised classification, the following techniques are often employed: soft independent modelling of class analogy (SIMCA) with PCA, K-nearest neighbours (KNN), linear discriminant analysis (LDA), Logistic regression, partial least squares discriminant analysis (PLSDA), and support vector machine classification [101]. Other unsupervised and supervised learning algorithms, which are independent component analysis (IDA), Fuzzy one class Support vector machines (FOCSVM), and associative classification, have also been described elsewhere [106,107,108,109]. It must be pointed out that for classification tasks, non-supervised learning approaches are not definitive in implementation but usually inform the analyst of a possible need for progressing into a more conclusive supervised learning methodology or peradventure stop moving ahead if the non-supervised learning results were deemed unsatisfactory. For any task with the end goal of classification (and not exploration), non-supervised learning technique should not stand alone unless used in conjunction with a supervised learning approach. However, supervised learning methodology is standard for classification task and could stand alone towards a conclusive analysis for a discrimination problem.

There are some present advances in integrating the pool of classifiers instead of using only single classifier for hyperspectral image classification. This approach is termed ensemble technique. With this technique, best use of classifiers’ strengths is optimized at the expense of the classifiers’ disadvantages [110,111,112]. There is likewise an increasing shift to deep learning globally, and application to the area of chicken egg fertility identification is not being left out. For example, [27] reported a classification study on 5 days incubation hatching eggs using methodology of transfer learning in conjunction with convolutional neural network (CNN). Overall accuracy of up to 99.5% was reported for a small-scale data set. A similar study by [113] used a multi-feature fusion approach based on transfer learning to classify 5–7days incubated chicken embryo eggs, and average accuracy of 98.4% was reported for small scale agricultural image samples. Apart from the fact that deep learning models from small scale data are not usually robust, there has been no research output to the best of our knowledge detailing the possibility of using this mentioned state-of-the art methodology for day 0 incubation egg data. Applied research efforts with this approach, therefore, remain at the basic levels, necessitating more in-depth studies.

#### 5.5.3. Performance Evaluation

Regression analysis performance is usually assessed in terms of the following criteria namely: correlation coefficient of calibration and validation (R_c_ and R_v_), coefficient of determination (R^2^), root mean square error of calibration and validation (RMSE_c_ and RMSE_v_), and predicted sum of squares (PRESS). While RMSE is a function of the model residuals, R^2^ can be understood as the fraction of the data information being explained by the model, and it is usually in close relation to correlation [102]. For classification, performance is usually evaluated in terms of the overall accuracy (OVA), which shows the overall percentage of correctly classified instances as against incorrectly classified instances. This criterion has, however, been regarded as misleading when considering the data set of an imbalanced nature [114], in which case, the choice of the confusion matrix evaluation criterion is considered more appropriate.

## 6. Hyperspectral Imaging for Chicken Egg Fertility Assessment

There have not been many works done on using hyperspectral imaging for chicken egg fertility assessment. The frequently occurring five published studies have been carried out by three notable research groups in the USA, Canada, and China [20,59,115,116,117]. In the work of [115], early fertility and embryonic development detection of hatching eggs were assessed in two separate experimental settings, using ratio wavelength 576/655 nm and ratio ranges between 576 nm and 682 ± 13 nm, for both brown and white eggs. While 12 eggs were imaged daily in two replicates and without replacement for four incubation periods in the first experimental set up for white eggs, 12 eggs were imaged daily in two replicates and with replacement for brown eggs in the second experimental set up. Experiment 1 outcome reported 1 of 46 fertile eggs detected for total eggs on days 0 and 1 incubation, 60% fertile and early embryonic development detection on day 2, and 91% fertility accuracy on day 3. Experiment 2 confirmed all considered eggs to be fertile upon breakout analysis and so fertility classification accuracy was impossible to be tested at this point. Apart from the fact that this work did not solve the early discrimination problem prior to incubation, the sample size was small, the replacement and non-replacement approaches for different types of eggs did not give a good basis for comparing performance of white and brown eggs, results validation was not done or reported, and it was not clear or stated explicitly the classification algorithm(s) used in the study.

A follow up hyperspectral imaging study similar to that of [115] was conducted by [20] on brown-shelled eggs in visible transmission wavelength regions of 420 to 840 nm. Both spectra and morphological attributes were considered in this study. Egg samples remained relatively the same as in the previous study, but Mahalanobis Distance Classification and PLSR algorithms were reportedly used for models’ development. Also, preprocessing operations of smoothening and multiplicative scatter correction were implemented. In the same vein, results were validated using leave one-out cross validation (LOV). Classification results of 100% on days 2 and 3, 95.8% on day 0, and 91.7% on day 1 were reported for Mahalanobis Distance (MD) Classifier. PLSR modelling algorithm on the other hand achieved 100% accuracy for all days of incubation, with LOV. Despite the presented results looking optimistic, breakout analysis showed there were no non-fertile eggs present in the sample size considered, and hence, the reported results were best regarded as being for egg embryonic development and not fertility. Likewise, there was no reported results upliftment with considering morphological attributes over spectra attributes. Therefore, fertility recognition problem prior to incubation at this juncture remains unsolved. The difficulty of having non-fertile eggs presence for training in a small sample size collection was thereby identified in this study. Future robust models will indeed need large data size for calibration, validation, and testing.

In another subsequent study by [116], the same 12 hatchery fertile eggs were used but now with matching up 12 non-fertile eggs acquired from flock raised without roosters. Hyperspectral images were then collected in eight replicates in the visible wavelength regions of 400 to 1000 nm. Adopting a MD Classifier, in conjunction with PCA, five replicates data were used for calibration and the remaining three replicates used for validation. A new set of three replications of 30 eggs each (of randomly mixed fertile and non-fertile eggs) was also reportedly used for verification. The outcome of this study presented overall accuracy for validation sets of eggs as 71% on day 0, 63% on day 1, 65% on day 2, and 83% on day 3, with lower verification results reported and 51% as the average. This study seems to be the first standard procedural set up to handle chicken egg fertility problem, using the non-destructive hyperspectral imaging technology. Notwithstanding, the sample size remains inadequate, and the study concluded from obtained results that the PCA/MD classifier adopted was inappropriate for early embryonic development and fertility detection. There is, therefore, a need for more appropriate modelling technique(s) to capture and learn accurately information from chicken egg fertility hyperspectral imaging data.

Liu and Ngadi [59] introduced the use of hyperspectral imaging technique in the near infrared (NIR) wavelength regions (900–1700 nm) to detect fertility and embryonic development in 174 white leghorn eggs. Mean spectral and image textural characteristics extracted using a Gabor filter algorithm were further analyzed in the study. The work finally implemented a dimensional reduction technique of PCA in conjunction with K-means clustering algorithm, towards model development. Best overall classification accuracies reported were 100% on day 0, 78.8% on day 1, 74.1% on day 2, 81.8% on day 3, and 84.1% on day 4. Hyperspectral imaging potential of determining fertility prior to incubation was therefore established in this study. Owing to the knowledge that improved the assessment of relevant chemical and other quality attributes that are more readily obtained in the NIR region, the success of Liu and Ngadi [59] was reportedly linked to higher wavelength consideration and improved HSI technology (including dimensional reduction techniques of PCA and feature extraction). Despite the great promising results obtained from this study, the modelling approaches adopted were non-supervised learning techniques, and so there is a need for further confirmation using standard supervised learning algorithm(s). Whereas some non-supervised learning approaches like the PCA, k-means, k-medians, and hierarchical cluster analysis are excellent with identifying/understanding grouping and clustering patterns in multidimensional data, they are limited when the end target is discrimination [118]. Furthermore, unsupervised classification is always the starting point in any discrimination problem and should necessarily be followed by supervised classification [119] towards an industrial adoptability consideration.

Similarly to earlier works, [117] compared the spectral and image morphological attributes of 90 green-shelled chicken egg towards hatchability detection. The study used a single selected optimum wavelength of 822 nm out of the considered visible wavelength regions between 400 to 1000 nm, and PCA in conjunction with Learning Vector Quantization Neural Network (LVQNN) were adopted as modelling algorithms. Overall accuracies using spectral characteristics were reported as 65% on day 0, 63% on day 1, 60% on day 2, 77% on day 3, and 83% on day 4. Results accuracy using morphological attributes were 72% on day 0, 70% on day 1, 76% on day 2, 97% on day 3, and 100% on day 4. The worthwhile improvement in accuracy brought about by using morphological attributes was only possible on days 3 and 4 incubation. Therefore, this study also did not solve the problem of fertility detection prior to incubation. The outcome of this study is, however, consistent with the earlier report of [20] that no textural or morphological attributes considered in the visible wavelength regions for brown eggs brought any significant improvement to models built from only spectral data.

A recent study by [120] reported a line-scan imaging analysis for fast viability recognition of fertilized egg embryo, with recorded overall recognition accuracy of up to 99%. The target in this work is more towards pharmaceutical application in which non-viable eggs can be useful for vaccine manufacture. Non-viability status of eggs was established by stopping incubation of randomly selected eggs on days 5 and 9, while the remaining fertilized eggs were kept incubated till day 13. These incubation days were already late for early fertility detection being sought for by the poultry industries. There was no predictive modelling exercise carried out in this study, and no standard supervised learning classifying algorithm was used. The work is therefore still preliminary towards solving the problem of egg fertility classification prior to incubation. From all the cases considered, it was only the work of [59] that showed the greatest potential of using non-destructive HSI technique for early fertility discrimination, especially prior to incubation, and this was possible considering the NIR wavelength regions of the light spectrum.

## 7. Future Works

Studies on the use of non-destructive technologies for chicken egg fertility assessment have evolved over the years. Hyperspectral imaging stood out as an emerging technology with the greatest potential among other non-destructive methods. Yet, the problem of fertility identification prior to incubation largely remains unresolved. Existing gaps in previous researches are related to data acquisition procedure, scanty available data, imbalanced data (too little or non-availability of non-fertile eggs for learning), and analysis/modelling techniques. Future works will need to use large enough quality data and embrace the use of existing approaches from the computing and biomedical allied fields for handling imbalanced data. Likewise, future studies should harness incorporating appropriately the state-of-the-art tools of machine and deep learning into the chicken egg fertility assessment modelling framework. Also, the use of ratio features in chicken egg fertility studies has not been adequately harnessed.

## 8. Conclusions

This review has focused on the present direction in the use of non-destructive technologies for food quality analysis, towards improvement in the use of non-destructive hyperspectral imaging technique for chicken egg fertility assessment. This paper reviewed existing non-destructive approaches including ultrasound and dielectric measurements, thermal imaging, machine vision, spectroscopy, and hyperspectral imaging. Promising results were more evident with machine vision, spectroscopy, and hyperspectral imaging than with other non-destructive techniques. The paper commenced by reviewing the present state of chicken egg production moved on to a discussion on egg formation and structure with exposition on chicken egg chemical and functional compositions. The intrinsic nature of chicken egg fertility and the industrial challenge of identifying fertility prior to incubation were examined. Existing methods of assessing chicken egg fertility were then investigated with a view to identifying existing problems and proffering a state-of-the-art solution (including appropriate technology) for solving such problems.

Three major areas needing attention towards building a futuristic robust model for chicken egg fertility early detection were eventually enunciated as sample size, analysis/modelling techniques, and the rare class data acquisition problem. Hyperspectral imaging technology was presented as adequate in solving the chicken egg fertility classification problem. Careful attention must, however, be given to acquiring good quality data with large sample size in respective categories and using appropriate analysis/modelling and evaluation techniques. It is believed that the appropriate implementation of the outcome of this review would tremendously assist the commercial hatchery industries towards achieving a stable and robust model for chicken egg fertility early discrimination.
